# Supplementary material for: Benthic Biodiversity, Carbon Storage and the Potential for Increasing Negative Feedbacks on Climate Change in Shallow Waters of the Antarctic Peninsula
Source: Biology (Basel). 2022 Feb 17;11(2):320. doi: 10.3390/biology11020320 (PMC8869673; doi:10.3390/biology11020320)
Supplement: Supplementary file 1 [file biology-11-00320-s001.zip › biology-1568740-supplementary.pdf]

Supplementary Material

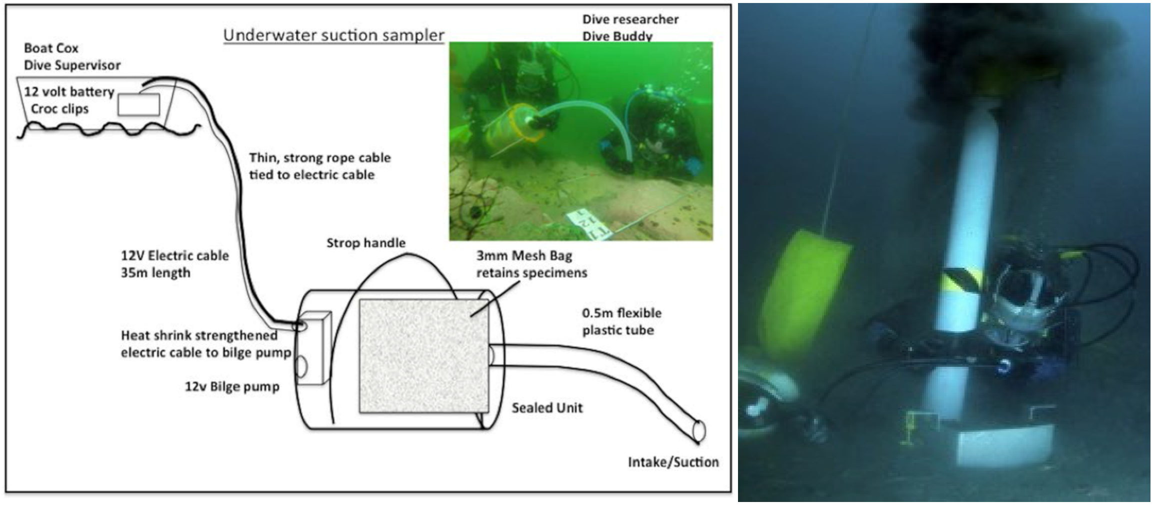

Fig. S1 Design of the battery operated suction sampler for rocky substrata (left panel; Souster, 2017) and the airlift suction sampler for soft substrata (right panel; Vause et al., 2019).

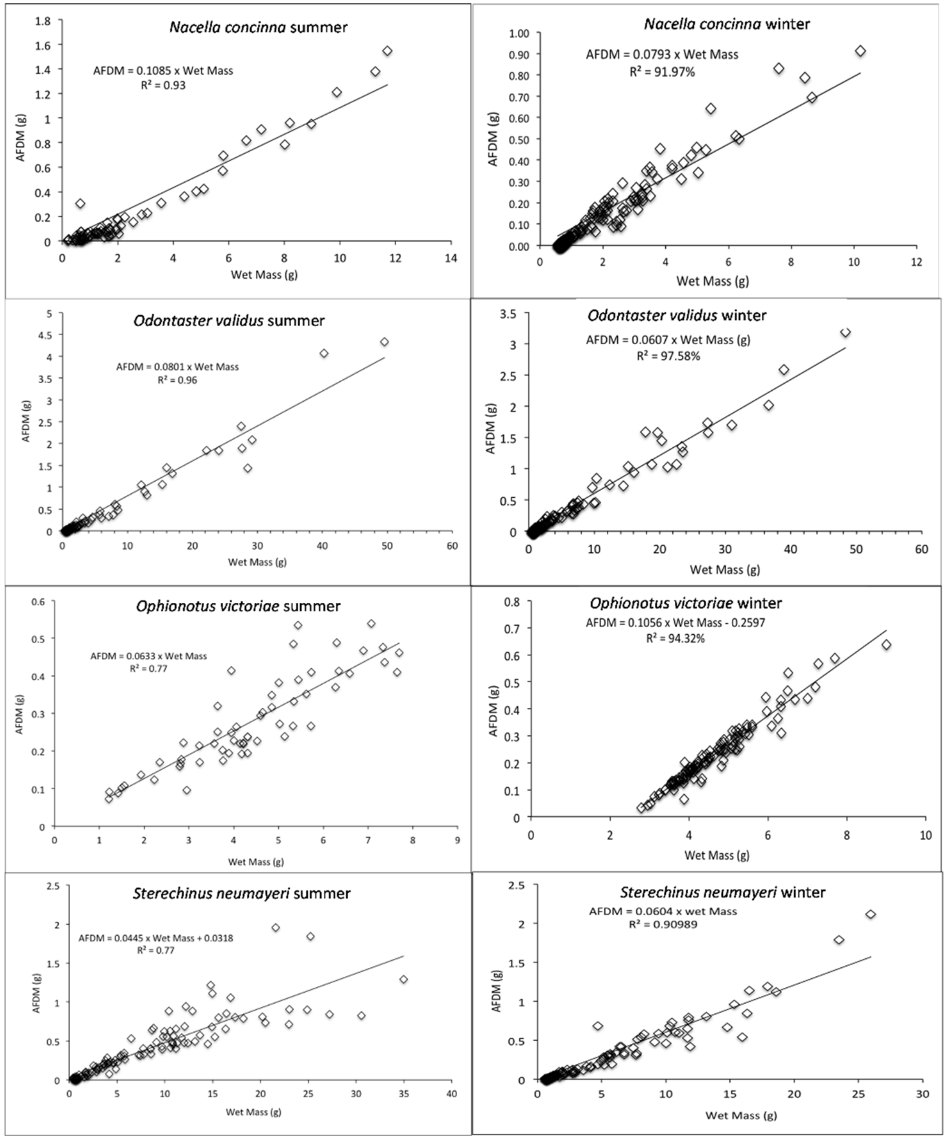

Fig S2. Morphometric relationships for hard substrata species that were used for mass conversions of individuals that were not weighed.

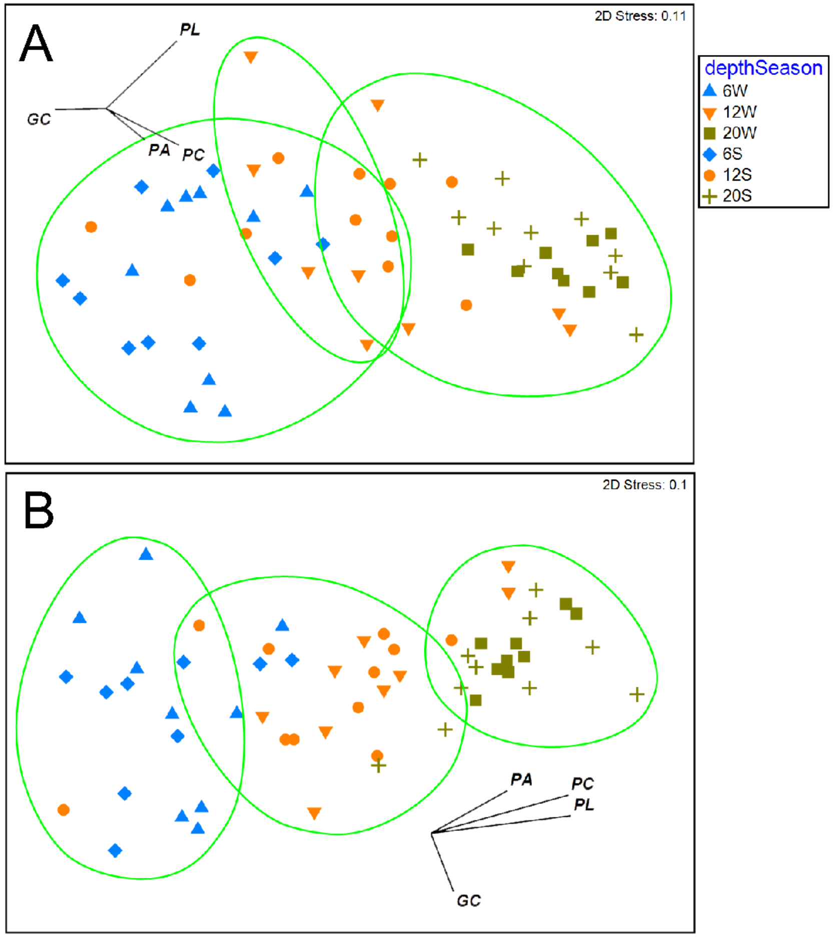

Fig S3. The similarity of carbon standing stock within the rocky shore functional groups around Rothera Point Antarctica, at 6, 12 and 20m in winter (W) and summer (S). A) organic (tissue) carbon m^{-2}, B) skeletal carbon, per m^{-2}. Groupings indicate similarity of A) 60% and B) 69%. All species were categorised into feeding guilds (see table), and in both cases the functional groupings driving the separation were GC (grazers), PL (mobile hard shelled scavenger/predator), PC (sessile hard shelled scavenger/predator) and PA (scavenger/predator arthropod).

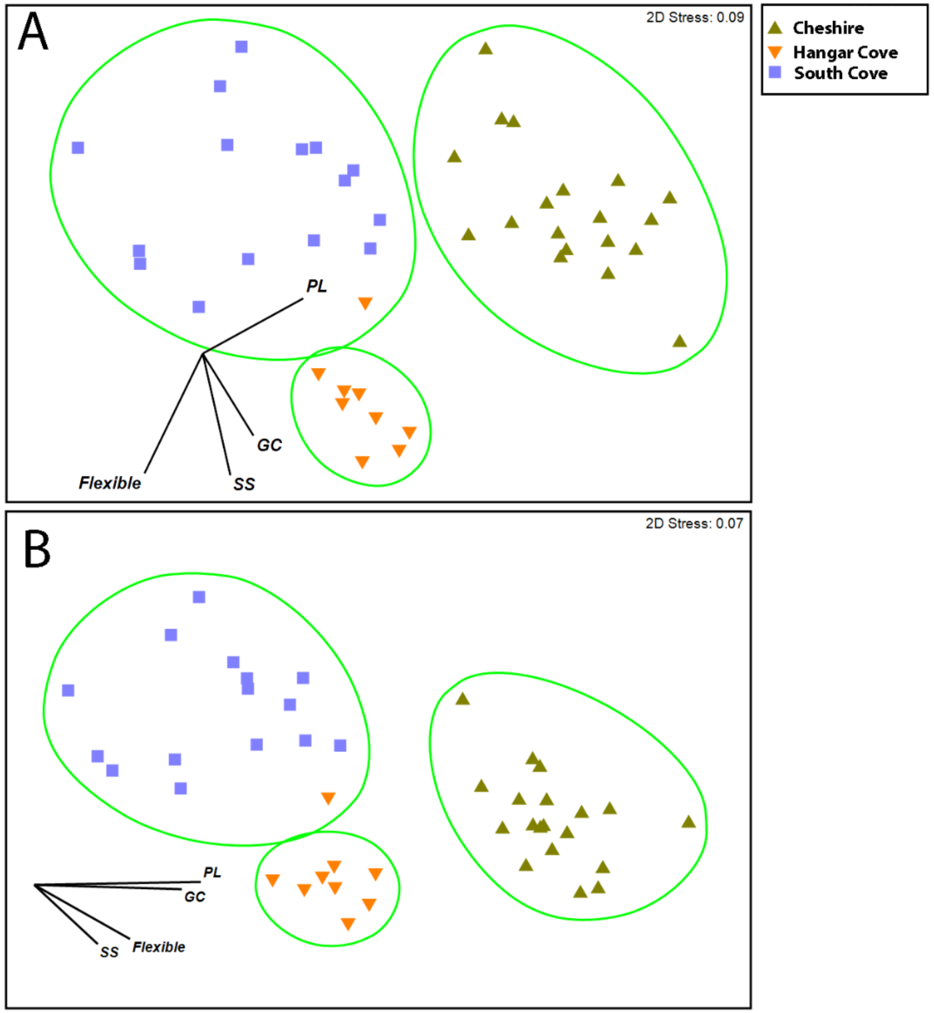

Figure S4. Comparison of carbon standing stock in A) organic tissues, m-2 and B) in skeletal tissues, m^{-2}, in both rocky (Cheshire) and soft (Hangar and South Cove) substratum functional groups sampled from 20m depth. Groupings indicate similarities of A) 63% and B) 68%. All species were categorised into feeding guilds (see table 1) and in both cases the functional groupings driving the separation were GC (grazers), PL (mobile hard shelled scavenger/predator), SS (sedentary suspension feeders) and dietary flexible species.

Table S1. Morphometric relationships for soft substrata species that were used for mass conversions of individuals that were not weighed. Location (Cove), Year and Season were included for species when they were significant factors in a PERMONOVA analysis. AFDM =ash free dry mass (g), AM = ash mass (g), L = length (mm)

Species	Cove	Year	Season	Equation
length to AFDM relationships				
Aequiyoldia eightsii	Hangar	2013	summer	$-0.00524 + 0.000017 L^3$
Aequiyoldia eightsii	South	2013	summer	$-0.0072 + 0.000019 L^3$
Aequiyoldia eightsii	Hangar	2014	summer	$-0.001796 + 0.000017 L^3$
Aequiyoldia eightsii	South	2014	summer	$-0.00766 + 0.000019 L^3$
Aequiyoldia eightsii	Hangar	2014	winter	$-0.002061 + 0.000015 L^3$
Aequiyoldia eightsii	South	2014	winter	$-0.0005 + 0.000017 L^3$
Aequiyoldia eightsii	Hangar	2015	summer	$-0.002352 + 0.000019 L^3$
Aequiyoldia eightsii	South	2015	summer	$-0.00506 + 0.000019 L^3$
Laternula elliptica	Hangar	2013	summer	$-0.066 + 0.000023 L^3$
Laternula elliptica	Hangar	2014	summer	$0.2092 + 0.000023 L^3$
Laternula elliptica	South	2014	summer	$0.0049 + 0.000025 L^3$
Laternula elliptica	Hangar	2014	winter	$0.182 + 0.000026 L^3$
Laternula elliptica	South	2014	winter	$-0.01609 + 0.000029 L^3$
Laternula elliptica	Hangar	2015	summer	$-0.233 + 0.000028 L^3$
Laternula elliptica	South	2015	summer	$-0.01073 + 0.000022 L^3$
Nacella concinna		2014		$-0.00208 + 0.000014 L^3$
Nacella concinna		2015		$0.0051 + 0.000014 L^3$
Ophionotus victoriae	South	2013	summer	$0.03081 + 0.000031 L^3$
Ophionotus victoriae	Hangar	2014	summer	$0.1912 + 0.000027 L^3$
Ophionotus victoriae	South	2014	summer	$0.02628 + 0.000034 L^3$
Ophionotus victoriae	Hangar	2014	winter	$-0.050 + 0.000042 L^3$
Ophionotus victoriae	South	2014	winter	$0.02740 + 0.000031 L^3$

Ophionotus victoriae	Hangar	2015	summer	0.189 + 0.000045 L^3
Ophionotus victoriae	South	2015	summer	-0.0843 + 0.000076 L^3
Sterechinus neumayeri	Hangar	2013		0.0306 + 0.000024 L^3
Sterechinus neumayeri	South	2013		0.0060 + 0.000018 L^3
Sterechinus neumayeri	Hangar	2014		-0.0326 + 0.000036 L^3
Sterechinus neumayeri	South	2014		0.0517 + 0.000029 L^3
Sterechinus neumayeri	Hangar	2015		0.0214 + 0.000037 L^3
Sterechinus neumayeri	South	2015		0.0468 + 0.000025 L^3
Length to DM relationships				
Laternula elliptica	Hangar	2013		0.000048 L^3
Laternula elliptica	Hangar	2014		0.000065 L^3
Laternula elliptica	South	2014		0.000061 L^3
Laternula elliptica	Hangar	2015		0.000067 L^3
Laternula elliptica	South	2015		0.000058 L^3
DM to AFDM relationships				
Aequiyoldia eightsii	Hangar	2013	summer	0.25433 DM
Aequiyoldia eightsii	South	2013	summer	0.27228 DM
Aequiyoldia eightsii	Hangar	2014	summer	0.26226 DM
Aequiyoldia eightsii	South	2014	summer	0.28821 DM
Aequiyoldia eightsii	Hangar	2014	winter	0.23809 DM
Aequiyoldia eightsii	South	2014	winter	0.25601 DM
Aequiyoldia eightsii	Hangar	2015	summer	0.28619 DM
Aequiyoldia eightsii	South	2015	summer	0.26350 DM
Laternula elliptica	Hangar	2013	summer	-0.182 + 0.5253 DM

Laternula elliptica	Hangar	2014	summer	0.4157 + 0.33440 DM
Laternula elliptica	South	2014	summer	-0.0055 + 0.4629 DM
Laternula elliptica	Hangar	2014	winter	-0.0431 + 0.38972 DM
Laternula elliptica	South	2014	winter	-0.004510 + 0.35195 DM
Laternula elliptica	Hangar	2015	summer	-0.3343 + 0.43525 DM
Laternula elliptica	South	2015	summer	-0.00182 + 0.36700 DM
Nacella concinna		2014		0.6577 DM
Nacella concinna		2015		0.6355 DM
Sterechinus neumayeri	Hangar	2013	summer	0.2915 DM
Sterechinus neumayeri	South	2013	summer	0.22255 DM
Sterechinus neumayeri	South	2014	summer	0.35353 DM
Sterechinus neumayeri	Hangar	2014		0.37113 DM
Sterechinus neumayeri	Hangar	2015	summer	0.36060 DM
Sterechinus neumayeri	South	2015	summer	0.31003 DM
Ophionotus victoriae	Hangar	2013	summer	0.2919 DM
Ophionotus victoriae	South	2013	summer	0.29235 DM
Ophionotus victoriae	South	2014	winter	0.29235 DM
Ophionotus victoriae	Hangar	2014	winter	0.3487 DM
Ophionotus victoriae	Hangar	2014	summer	0.32858 DM
Ophionotus victoriae	South	2014	summer	0.28832 DM
Ophionotus victoriae	Hangar	2015	summer	0.4381 DM
Ophionotus victoriae	South	2015	summer	0.3786 DM

Table S2. Dive site information collated from Antarctic SCUBA diving practitioners.

Location Name	LAT(DD)	LONG(DD)	Substratum	Topography	Comments
Dumbbell Island in the Terra Firma Islands	-68.69208	-67.52688	hard	Hard Rock Community	American Gould Trip 2019
Stonington Island	-68.18333	-67.00000	hard	rocky	Lisa Trotter Dive Guide
Randall Rocks off Millerand Island	-68.17577	-67.26823	hard	Hard Rock Community	American Gould Trip 2019
Troval	-67.77888889	-68.42111111	hard	hard	Carlos Angulo, Spain
Carvahal	-67.766525	-68.8962	hard		Silvia Murcia, Inach
Mikkelsen Island	-67.63333333	-68.18333333	hard	hard bottom	Ricardo Sahade Sedna sail ship dive sites
Small island between Pourqoui Pas and Blaiklock Islands	-67.55667	-67.24722	hard	Hard Rock Community	American Gould Trip 2019
Small island off a small island to the north of Pinero Island	-67.54882	-67.77137	hard	Hard Rock Community	American Gould Trip 2019
Tutton Point on Laird Island	-66.87732	-67.57520	hard	Hard Rock Community	American Gould Trip 2019
Detaille Island	-66.86819167	-66.79431111	hard	Ice Scourded Rocky Shelves	Lisa Kelly IAATO
Detaille Island	-66.86739722	-66.78811667	hard	Rocky wall, Ice Scoured for top 20 m	Lisa Kelly IAATO
Detaille Island	-66.86667	-66.50000	hard	rocky	Lisa Trotter Dive Guide
Southern Saffery Islands	-66.08942	-65.83855	hard	Hard Rock Community	American Gould Trip 2019
Fish Island	-66.03333333	-65.41666667	hard	hard bottom	Ricardo Sahade Sedna sail ship dive sites
Minnow Islands (part of Fish Islands South of Prospect Point)	-66.02505	-65.35333	hard	Hard Rock Community	American Gould Trip 2019
Prospect Point	-66.01667	-65.35000	hard	rocky	Lisa Trotter Dive Guide

Cuverville Island	-66.00440	-65.36052	mixed	gravel	Ashley Knight and Peter Webster Nat Geo
Small island south of Markham Island	-65.94492	-66.02482	hard	Hard Rock Community	American Gould Trip 2019
Renaud Island	-65.88222222	-66.09361111	hard	hard bottom	Ricardo Sahade Sedna sail ship dive sites
Berthelot Islands, Grandidier Channel	-65.52528	-64.20639	hard	rocky and gravely bottom	Beligica 120 Expedition 2019
Small island to the norht of Lahille Island and south of Lippmann Island	-65.51312	-64.42027	hard	Hard Rock Community	American Gould Trip 2019
Galindez Island	-65.24689	-64.24413	hard	Boulder Shores	Dkab sites from Barnes Arnold 2001 paper and I extracted coordinated from google maps
Vernadsky, Wall near channel entrance	-65.24610	-64.22780	hard	rocky reef	Ashley Knight and Peter Webster Nat Geo
Vernadsky, Argentine Islands, Corner Island	-65.24498	-64.24123	hard	rocky reef	Ashley Knight and Peter Webster Nat Geo
Vernadsky	-65.24446	-64.24258	soft	short rock wall down to soft bottom	Gail Ashton Peninsula Dives
Argentine Islands near Vernadsky	-65.24018	-64.23087	hard	Hard Rock Community	American Gould Trip 2019
Petermann Island	-65.16667	-64.16667	hard	rocky	Lisa Trotter Dive Guide
Petermann Island	-65.14453333	-64.09897778	hard	Rocky Shelves mainly scoured top 20 m	Lisa Kelly IAATO
Hovgaard Islands	-65.11583	-64.34222	soft	highly protected and almost enclosed inner sandy bay	Beligica 120 Expedition 2019
Pleneau Island (just off penguin colony on SE corner)	-65.10425	-64.04705	hard	Hard Rock Community	American Gould Trip 2019
Pleneau Island	-65.10000	-64.06667	hard	boulders and pebbles	Lisa Trotter Dive Guide
Punta Gutierrez	-65.08555556	-63.13888889	soft	soft	Carlos Angulo Spain

Booth Island	-65.08333	-64.00000	hard	rocky	Lisa Trotter Dive Guide
Booth Island	-65.07495556	-64.00599167	mixed	Rocky with areas of Ice Scour and Soft Substrate	Lisa Kelly IAATO
Wauwermans Island #514	-64.91668	-64.03492	hard	hard rock	Sabrina Heiser Alabama University
Skontorp Cove, Paradise Harbour	-64.90528	-63.08472	soft	highly protected inner cove with muddy bottoms	Beligica 120 Expedition 2019
Wauwermans Islands	-64.90017	-63.85313	hard	Hard Rock Community	American Gould Trip 2019
Paradise Bay, Shag Wall	-64.89760	-62.87250	hard	rock wall	Ashley Knight and Peter Webster Nat Geo
Paradise Bay, Shag Cliffs	-64.89452	-62.90429	hard	rock wall	Ashley Knight and Peter Webster Nat Geo
Paradise Bay, Under Bird Cliffs	-64.89442	-62.87157	hard	rocky wall with gravel/silt on slopes	Ashley Knight and Peter Webster Nat Geo
Paradise Bay	-64.88333333	-62.88333333	hard	hard bottom	Ricardo Sahade Sedna sail ship dive sites
Peltier Channel	-64.86493056	-63.51063611	hard	Rocky	Lisa Kelly IAATO
Paradise Harbor	-64.85000	-62.90000	hard	rocky	Lisa Trotter Dive Guide
Neko Harbour	-64.84560	-62.52880	soft	sand/fine silt	Ashley Knight and Peter Webster Nat Geo
Paradise Harbour	-64.84435556	-62.86737222	mixed	Soft Substrate with Rocks Interspersed	Lisa Kelly IAATO
Port Lockroy, Peltier channel	-64.83002	-63.49547	mixed	rocky reef with silt on slopes	Ashley Knight and Peter Webster Nat Geo
Port Lockroy, Jougla point	-64.82790	-63.49570	soft	silt	Ashley Knight and Peter Webster Nat Geo
Port Lockroy, GoPro Site - RIP	-64.82738	-63.49553	mixed	Silty with rocky protrusions	Ashley Knight and Peter Webster Nat Geo

Port Lockroy, Jougla Point	-64.82710	-63.49231	soft	silt	Ashley Knight and Peter Webster Nat Geo
Port Lockroy, Portside Rocks	-64.82710	-63.49231	Mixed	rocky reef with silt on slopes	Ashley Knight and Peter Webster Nat Geo
Jougla Point, Swallow Whale Bones	-64.82607	-63.49225	soft	silt	Ashley Knight and Peter Webster Nat Geo
Port Lockroy	-64.82588333	-63.48757222	mixed	Glacial Silt with Rocky slopes	Lisa Kelly IAATO
Port Lockroy, Between Base and Glacier	-64.82550	-63.48882	soft	very fine silt	Ashley Knight and Peter Webster Nat Geo
Paradise Bay	-64.82398056	-62.85893056	hard		Silvia Murcia was Inach
Paradise Harbor	-64.82083	-62.85500	hard	rock wall	Ashley Knight and Peter Webster Nat Geo
Dobrowolski Island	-64.80835	-63.30737	hard	Boulder Shores	Dkab sites from Barnes Arnold 2001 paper and I extracted coordinated from google maps
North Laggard Island Wall	-64.80598	-64.01578	hard	wall + hard rock	Sabrina Heiser Alabama University
Limitrophe Island	-64.79320	-64.00723	hard	Hard Rock Community	American Gould Trip 2019
Limitrophe Island	-64.79277	-64.99845	hard	hard rock	Sabrina Heiser Alabama University
Joubin Island #1 (Howard Island)	-64.78830	-64.36222	hard	hard rock	Sabrina Heiser Alabama University
Stepping Stones	-64.78378	-63.99107	hard	hard rock	Sabrina Heiser Alabama University
Port Lockroy	-64.78333	-64.10000	hard	rocky	Lisa Trotter Dive Guide
Bahia Paraiso	-64.78045	-64.06250	hard	hard rock + ship wreck	Sabrina Heiser Alabama University

SE corner of Bonaparte Point, Anvers Island	-64.77927	-64.04408	hard	Hard Rock Community	American Gould Trip 2019
SE Bonaparte Point	-64.77912	-64.04190	hard	hard rock + occasionally sediment	Sabrina Heiser Alabama University
Anvers Island	-64.775	-64.06666667	mixed	mixed	Ricardo Sahade Sedna sail ship dive sites
Palmer Pier	-64.77458	-64.05458	hard	hard rock + man made pier	Sabrina Heiser Alabama University
Beaumont Skerries	-64.77448	-64.30925	hard	walll	Sabrina Heiser Alabama University
Gamage Point	-64.77407	-64.05665	hard	hard rock + some sediment at bottom	Sabrina Heiser Alabama University
Joubin Islands	-64.77195	-64.36985	hard	Hard Rock Community	American Gould Trip 2019
East Litchfield Island	-64.76853	-64.08385	hard	hard rock + some sediment at bottom	Sabrina Heiser Alabama University
Old Palmer	-64.76522	-64.07808	hard	hard rock	Sabrina Heiser Alabama University
Norsel Point	-64.76045	-64.09833	hard	wall + hard rock	Sabrina Heiser Alabama University
Useful Island	-64.75722	-62.91083	hard	rocky shallows to muddy substrate with gravels at depth	Beligica 120 Expedition 2019
Danco	-64.74537	-62.65826		stunning rock wall (prob best dive site for me in Antarctica	Gail Ashton Peninsula Dives
Danco Island, Ronge Island	-64.74420	-62.65535	hard	sheer wall dropping virtually to 50m - ROV footage nearby shows bouldery seabed	Ashley Knight and Peter Webster Nat Geo
Green Reef. Neumayer Channel	-64.73306	-63.53722	soft	Soft sand, muddy	Beligica 120 Expedition 2019

Casey Islands	-64.72805	-64.24857	hard	hard rock	Sabrina Heiser Alabama University
Dream Island	-64.72397	-64.22313	hard	hard rock	Sabrina Heiser Alabama University
Cuverville Island	-64.68333	-62.63333	hard	rocky	Lisa Trotter Dive Guide
Cuverville Island East Wall	-64.68313056	-62.61328056	hard	Rock Wall, Scoured to 10 M then rock with Kelp	Lisa Kelly IAATO
Cuverville, shallow spit	-64.68085	-62.62058	mixed	pebbley (~5-10cm)	Ashley Knight and Peter Webster Nat Geo
Cuverville Wall	-64.68010	-62.61710	hard	rocky reef with gravel	Ashley Knight and Peter Webster Nat Geo
Cueverville	-64.67990	-62.61853	mixed	gravel	Ashley Knight and Peter Webster Nat Geo
Opposite Side of Orne Harbour	-64.64852222	-62.56698056	hard	Ice Scoured, Shelving Wall	Lisa Kelly IAATO
Orne Harbour	-64.63286111	-62.54429167	hard	Wall	Lisa Kelly IAATO
Enterprise Island, Governoren wreck	-64.54070	-61.99820	mixed	silt surrounding wreck	Ashley Knight and Peter Webster Nat Geo
Foyn Harbour, Enterprise Island, Governoren wreck	-64.54023	-62.00135	mixed	silt surrounding wreck	Ashley Knight and Peter Webster Nat Geo
Portal Point, Lone rock near spit	-64.49740	-61.76480	hard	~30cm round rocks	Ashley Knight and Peter Webster Nat Geo
Melchoir Island	-64.41111111	-62.87611111	hard	hard bottom	Ricardo Sahade Sedna sail ship dive sites
Isla Musgo	-64.38694444	-61.21972222	hard	rock wall	Carlos Angulo Spain
Melchior Island	-64.38500	-63.02083	mixed	muddy bottom with gravels and dropstones	Beligica 120 Expedition 2019

Cierva Cove	-64.15000	-60.85500	hard	rocky/bouldery	Ashley Knight and Peter Webster Nat Geo
Hydruga Rocks	-64.13333	-61.91667	soft	sand	Lisa Trotter Dive Guide
Mikkelsen Harbor (D'Hainaut)	-63.90542778	-60.79363333	hard	Rocky with Kelp	Lisa Kelly IAATO
Punta Sheppard Point	-63.8775	-56.98666667	soft	soft	Carlos Angulo Spain
Aitcho Island, Barrientos	-63.86843	-60.97487	hard	rocky reef	Ashley Knight and Peter Webster Nat Geo
Spert Island	-63.86200	-60.98580	Hard	surgey channel with rock walls, boulders and sand	Ashley Knight and Peter Webster Nat Geo
Paulet Island	-63.58333	-55.78333	hard	rocky	Lisa Trotter Dive Guide
Paulet Island	-63.57400833	-55.79905833	mixed	Ice Scourded Pebbly Bottom	Lisa Kelly IAATO
Paulet Island	-63.57040	-55.77430	soft	silt	Ashley Knight and Peter Webster Nat Geo
Paulet Island	-63.57040	-55.77430	hard	round rocks ~30cm	Ashley Knight and Peter Webster Nat Geo
Paulet Island	-63.56891944	-55.80741667	mixed	Ice Scourded Pebbly Bottom	Lisa Kelly IAATO
Brown Bluff	-63.52153889	-56.879025	hard	Iceberg Scour and Rocky Substrate	Lisa Kelly IAATO
Brown Bluff, Rock at South End	-63.51057	-56.90540	mixed	sand/gravel	Ashley Knight and Peter Webster Nat Geo
Brown Bluff	-63.51037	-56.87507	soft	sand/fine silt	Ashley Knight and Peter Webster Nat Geo
Esperanza Bay	-63.41027778	-56.99527778	mixed	mixed	Ricardo Sahade Sedna sail ship dive sites
Tay Head	-63.35260278	-55.55168056	hard	Ice Scoured Rock	Lisa Kelly IAATO

Base O higgins	-63.32053333	-57.89756667	hard		Silvia Murcia was Inach
BAE Gabriel de castilla	-63.12972222	-60.82777778	soft	soft	Carlos Angulo Spain
Deception Island, Neptune's Bellows	-62.99210	-60.55155	hard	sheer cliffs/rock	Ashley Knight and Peter Webster Nat Geo
Deception Island	-62.99083056	-60.56027222	hard		Silvia Murcia was Inach
Deception Island, Whalers Bay	-62.98990	-60.55930	Mixed	gravel/volcanic ash with sheer rock faces from 27m. *Site is inside a caldera	Ashley Knight and Peter Webster Nat Geo
Deception Island Whaler's Bay	-62.98744444	-60.55296111	hard	Wall, Rocky with Kelp; plateaus with Volcanic Cinder.	Lisa Kelly IAATO
Deception Island Whalers's Bay	-62.98336667	-60.56410833	hard	Volcanic cinder	Lisa Kelly IAATO
Deception Island Port Foster	-62.95110278	-60.63736111	hard	Volcanic cinder	Lisa Kelly IAATO
Deception Island	-62.93527778	-60.60444444	soft	soft bottom	Ricardo Sahade Sedna sail ship dive sites
Deception Island	-62.92406111	-60.67218333	hard		Silvia Murcia was Inach
Punta SW	-62.83555556	-60.10638889	hard	hard	Carlos Angulo Spain
Rackelias rocks	-62.79916667	-60.51666667	soft	soft	Carlos Angulo Spain
Snow Island	-62.74241	-61.47604	hard	Boulder Shores	Dkab sites from Barnes Arnold 2001 paper and I extracted coordinated from google maps

Livingston Island	-62.65888889	-60.36472222	soft	soft bottom	Ricardo Sahade Sedna sail ship dive sites
Punta Hanna	-62.65436111	-60.61175	hard		Silvia Murcia was Inach
Islote Albatros	-62.65194444	-58.94638889	hard	hard	Carlos Angulo Spain
Neko Harbor	-62.59717	-59.89167	soft	sand/fine silt	Ashley Knight and Peter Webster Nat Geo
Punta NW	-62.58388889	-59.96194444	hard	Rocky wall	Carlos Angulo Spain
Half Moon Island	-62.58210	-59.91840	mixed	rocky to around 15m then gravel to fine sediment by 30m	Ashley Knight and Peter Webster Nat Geo
Arturo Base	-62.47105	-59.62871667	hard		Silvia Murcia was Inach
Aitcho Islands	-62.41667	-59.71667	hard	rocky	Lisa Trotter Dive Guide
Lemaire Channel, Barrientos Island	-62.41442	-59.73480	mixed	sand/gravel with rocky reefs	Ashley Knight and Peter Webster Nat Geo
Barrientos	-62.41430	-59.73460	hard	rocky reef	Ashley Knight and Peter Webster Nat Geo
Cecelia Island	-62.41256111	-59.71913889	hard	Rocky Substrate with Kelp	Lisa Kelly IAATO
Islotes E Artigas	-62.26333333	-58.94444444	hard	hard	Carlos Angulo Spain
KGI	-62.21983333	-58.78083333	soft		Ahn (Korea) KGI
ZA-28	-62.21078	-58.41977	mixed	Mixed (mainly soft)	Piotr Balazy (Poland DO)
KGI	-62.21066667	-58.73916667	soft		Ahn (Korea) KGI

KGI	-62.20883333	-58.7345	soft		Ahn (Korea) KGI
KGI	-62.20483333	-58.72383333	soft		Ahn (Korea) KGI
ZA-29	-62.19755	-58.42842	mixed	Mixed (mainly soft)	Piotr Balazy (Poland DO)
ZA-6	-62.18265	-58.61058	mixed	Mixed (mainly soft)	Piotr Balazy (Poland DO)
ZA-17	-62.18120	-58.58837	mixed	Mixed (mainly soft)	Piotr Balazy (Poland DO)
ZA-4	-62.18015	-58.53433	mixed	Mixed (mainly soft)	Piotr Balazy (Poland DO)
ZA-13	-62.17802	-58.53385	mixed	Mixed (mainly soft)	Piotr Balazy (Poland DO)
ZA-5	-62.17712	-58.54568	mixed	Mixed (mainly soft)	Piotr Balazy (Poland DO)
ZA-14	-62.17415	-58.62298	mixed	Mixed (mainly soft)	Piotr Balazy (Poland DO)
ZA-3	-62.17007	-58.51725	mixed	Mixed (mainly soft)	Piotr Balazy (Poland DO)
ZA-23	-62.16883	-58.56025	mixed	Mixed (mainly soft)	Piotr Balazy (Poland DO)
ZA-18	-62.16702	-58.54493	mixed	Mixed (mainly soft)	Piotr Balazy (Poland DO)
ZA-33	-62.16673	-58.44010	hard	Rock	Piotr Balazy (Poland DO)
ZA-15	-62.16458	-58.56385	mixed	Mixed (mainly soft)	Piotr Balazy (Poland DO)
ZA-32	-62.16450	-58.56115	mixed	Mixed (mainly soft)	Piotr Balazy (Poland DO)
ZA-22	-62.16422	-58.55337	mixed	Mixed (mainly soft)	Piotr Balazy (Poland DO)
ZA-10	-62.16418	-58.55122	mixed	Mixed (mainly soft)	Piotr Balazy (Poland DO)
ZA-19	-62.16253	-58.50208	mixed	Mixed (mainly soft)	Piotr Balazy (Poland DO)
ZA-12	-62.16060	-58.59377	mixed	Mixed (mainly soft)	Piotr Balazy (Poland DO)
ZA-7	-62.15992	-58.59167	mixed	Mixed (mainly soft)	Piotr Balazy (Poland DO)
ZA-11	-62.15867	-58.59752	mixed	Mixed (mainly soft)	Piotr Balazy (Poland DO)
ZA-1	-62.15827	-58.45868	mixed	Mixed (mainly soft)	Piotr Balazy (Poland DO)
ZA-16	-62.15480	-58.55257	mixed	Mixed (mainly soft)	Piotr Balazy (Poland DO)
ZA-8	-62.15377	-58.54560	mixed	Mixed (mainly soft)	Piotr Balazy (Poland DO)
ZA-9	-62.15315	-58.54143	mixed	Mixed (mainly soft)	Piotr Balazy (Poland DO)
ZA-20	-62.14872	-58.52710	mixed	Mixed (mainly soft)	Piotr Balazy (Poland DO)

ZA-2	-62.14150	-58.50748	mixed	Mixed (mainly soft)	Piotr Balazy (Poland DO)
ZA-21	-62.14100	-58.50397	hard	Rock	Piotr Balazy (Poland DO)
ZA-31	-62.12532	-58.39678	mixed	Mixed (mainly soft)	Piotr Balazy (Poland DO)
ZA-30	-62.11705	-58.39475	mixed	Mixed (mainly soft)	Piotr Balazy (Poland DO)
Mackellar Fjord KGI	-62.10450	-58.45860	hard	Hard (cobbles)	Video footage of the sites available
Martel Inlet KGI	-62.10040	-58.33260	mixed	Mixed (mainly soft)	Video footage of the sites available
Martel Inlet KGI	-62.10000	-58.33120	hard	Hard (boulders, cobbles)	Video footage of the sites available
Martel Inlet KGI	-62.09800	-58.32970	hard	Hard (boulders, cobbles and pebbles)	Video footage of the sites available
Martel Inlet KGI	-62.09680	-58.33230	mixed	Mixed (mainly soft)	Video footage of the sites available
Mackellar Fjord KGI	-62.09240	-58.41040	hard	Hard (cobbles)	Video footage of the sites available
ZA-24	-62.09133	-58.48763	mixed	Mixed (mainly soft)	Piotr Balazy (Poland DO)
Mackellar Fjord KGI	-62.09090	-58.46520	hard	Hard (cobbles and boulders), no slope	Video footage of the sites available
Mackellar Fjord KGI	-62.09060	-58.47300	hard	Hard (boulders, cobbles), pronounced slope	Video footage of the sites available
Mackellar Fjord KGI	-62.08980	-58.45160	mixed	Mixed	Video footage of the sites available
Mackellar Fjord KGI	-62.08050	-58.46350	mixed	Mixed	Video footage of the sites available
ZA-25	-62.07958	-58.42727	mixed	Mixed (mainly soft)	Piotr Balazy (Poland DO)
Mackellar Fjord KGI	-62.07780	-58.46030	mixed	Mixed	Video footage of the sites available
ZA-26	-62.07600	-58.35560	mixed	Mixed (mainly soft)	Piotr Balazy (Poland DO)

ZA-27	-62.07287	-58.39460	mixed	Mixed (mainly soft)	Piotr Balazy (Poland DO)
South Shetlands, Point Wild, Elephant Island, Inside wall of point	-61.09610	-54.85950	mixed	gravel/sand between rocky outcrops	Ashley Knight and Peter Webster Nat Geo
Anchor. D Plates	-67.5931166	-68.1897333	hard		
Cheshire Island	-67.5733833	-68.1255	hard		
East Beach	-67.5695833	-68.11335	mixed		
Back Bay Lagoon	-67.5952666	-68.2532833	mixed		
Rose Garden	-67.6118833	-68.2094833	hard		
Leonie Island	-67.6049333	-68.3209833	hard		
Mackay Dive	-67.5363665	-68.07235	hard		
Shack's Crack	-67.5545833	-68.1971833	hard		
Trolval	-67.5954666	-68.2100333	hard		
Temporary Jetty	-67.5688	-68.1301165	mixed		
- Wharf 2019	-67.57195	-68.1297166	hard		
Mackay Point	-67.5388833	-68.0758	hard		
Killingbeck 1	-67.5712333	-68.0691666	hard		
Lagoon	-67.5938666	-68.24245	mixed		
Leonie NE	-67.5925	-68.33975	hard		
Killingbeck 2	-67.5720333	-68.0712833	hard		
Killingbeck 3	-67.5733666	-68.0756	hard		
S Cove Logger	-67.5708666	-68.1327333	hard		
Sediment C 20m	-67.5692166	-68.1340833	soft		
T1 5m	-67.5702666	-68.13245	hard		
Killingbeck	-67.5756333	-68.1059666	hard		HAZ025
Killingbeck	-67.5708333	-68.0924	hard		HAZ021
Killingbeck	-67.5749833	-68.08275	hard		HAZ030
Killingbeck	-67.5797166	-68.0836999	hard		HAZ036
Killingbeck	-67.5757666	-68.0989666	hard		HAZ027

Macay Point	-67.5386166	-68.0651166	hard		HAZ008
Leonie Pinnacle 1	-67.5846166	-68.3446666	hard		HAZ046
Leonie Pinnacle 2	-67.5793	-68.3151	hard		HAZ005
HAZ044	-67.61335	-68.3248166	hard		
Kirsty Brown	-67.5968333	-68.2630833	hard		
Donnelly	-67.61055	-68.2027666	hard		
Hangar Cove	-67.564278	-68.127347	soft		
North Cove	-67.566071	-68.120758	soft		

Table S3. General Linear Model (Minitab 19) the relationship between the number of functional groups and carbon standing stock.

Factor	Type	Levels	Values		
Cove	Fixed	2	Hangar, South		
Substrata	Fixed	2	rocky, soft		
Season	Fixed	2	summer, winter		
Analysis of variance					
Source	DF	Adj SS	Adj MS	F - Value	P – Value
Log(10) Functional Groups	1	0.30	0.30	6.28	0.02
Log(10) Functional Groups^2	1	0.58	0.58	11.96	<0.01
Cove	1	1.58	1.58	32.49	<0.01
Substrata	1	4.68	4.68	96.34	<0.01
Season	1	0.02	0.02	0.34	0.56
Error	69	3.35	0.05		
Total	74	12.27			
